# Optical Marker-Based Motion Capture of the Human Spine: A Scoping Review of Study Design and Outcomes

**DOI:** 10.1007/s10439-024-03567-0

**Published:** 2024-07-18

**Authors:** Claudia F. Romero-Flores, Rogelio Bustamante-Bello, Marcos Moya Bencomo, Erick Axel Martinez-Ríos, Luis Montesinos

**Affiliations:** Tecnologico de Monterrey, School of Engineering and Sciences, Ave. Eugenio Garza Sada 2501, Monterrey, N.L. México 64849

**Keywords:** Spine biomechanics, Thoracolumbar spine, Optical Motion Capture

## Abstract

Biomechanical analysis of the human spine is crucial to understanding injury patterns. Motion capture technology has gained attention due to its non-invasive nature. Nevertheless, traditional motion capture studies consider the spine a single rigid segment, although its alignment changes during movement. Moreover, guidelines that indicate where markers should be placed for a specific exercise do not exist. This study aims to review the methods used to assess spine biomechanics using motion capture systems to determine the marker sets used, the protocols used, the resulting parameters, the analysed activities, and the characteristics of the studied populations. PRISMA guidelines were used to perform a Scoping Review using SCOPUS and Web of Science databases. Fifty-six journal and conference articles from 1997 to 2023 were considered for the analysis. This review showed that Plug-in-Gait is the most used marker set. The lumbar spine is the segment that generates the most interest because of its high mobility and function as a weight supporter. Furthermore, angular position and velocity are the most common outcomes when studying the spine. Walking, standing, and range of movement were the most studied activities compared to sports and work-related activities. Male and female participants were recruited similarly across all included articles. This review presents the motion capture techniques and measurement outcomes of biomechanical studies of the human spine, to help standardize the field. This work also discusses trends in marker sets, study outcomes, studied segments and segmentation approaches.

**Graphical Abstract:**

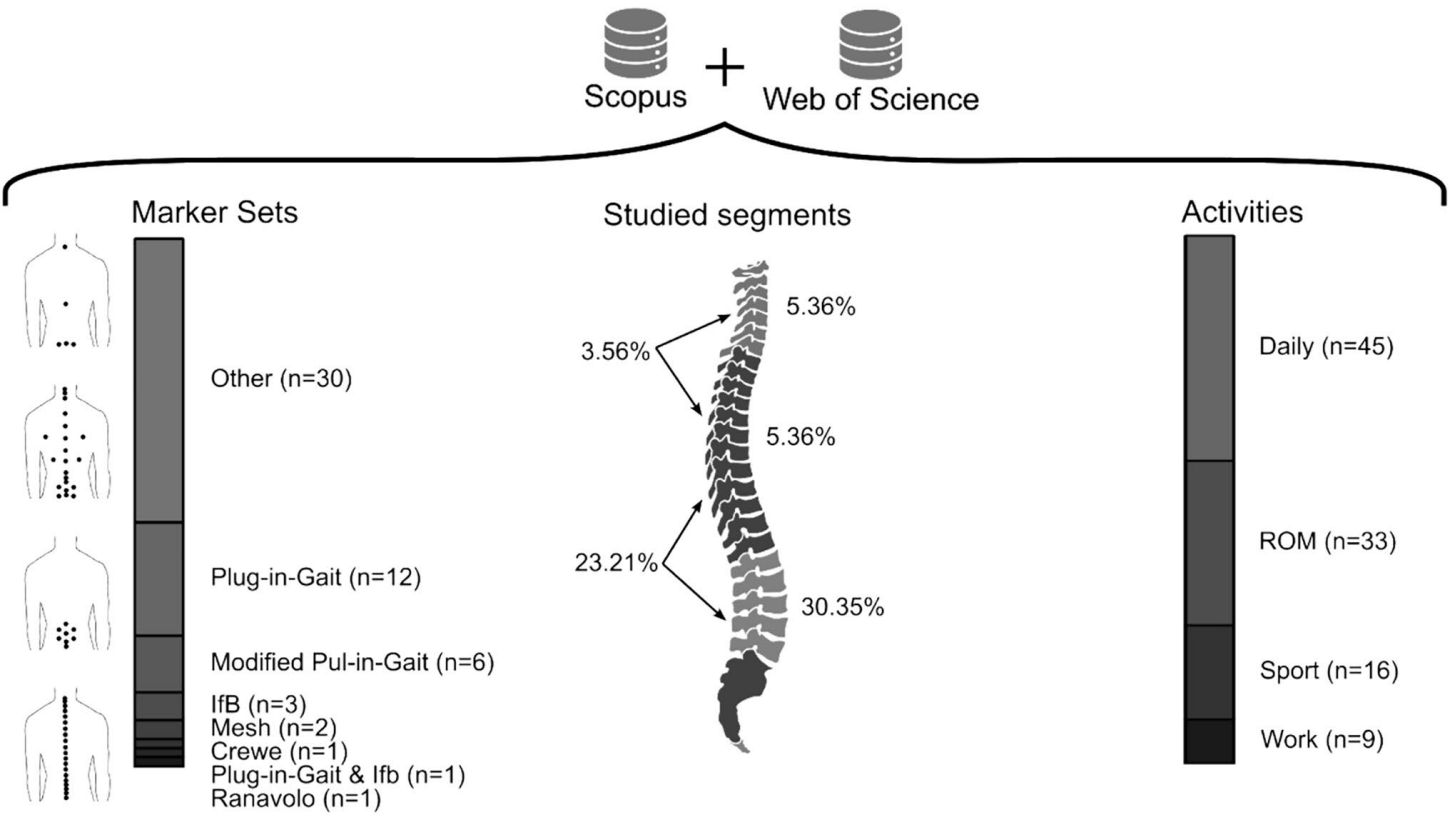

## Introduction

The human spine is a complex and primary structure of the human body. The spine consists of thirty-three individual vertebrae in humans, separated by the intervertebral discs. Across populations, the spine has various shapes, lengths, alignments, and ranges of motion (ROM). Musculoskeletal models represent the body as a combination of rigid segments and mechanical joints. However, the torso does not behave naturally as either type. This complexity makes the detailed study of the movement a big challenge. Investigating motion and loading patterns might help to understand pathologies and injury mechanisms [1–3]. For instance, altered spinal kinematics have been detected in patients suffering from spine pain [4]. Furthermore, every sports discipline has the risk of developing spine injuries, especially in the lumbar area [5]. Overuse injuries are usually caused by repetitive movement combined with high loading and lack of rest [6]. Thus, adequate monitoring of the spine motion is crucial to detect injuries in the early stages.

In traditional biomechanical studies, the spine and torso are considered rigid segments for kinematics and kinetics analysis [6]. Recent studies have attempted to do a multi-segmental analysis of the spine. This information is crucial to understanding the relationship between internal loadings and injuries in spine structures, such as intervertebral discs. Recent studies have found significant differences in kinematics and kinetics between populations using a multi-segment rather than a single-segment approach [6–8]. Kudo et al. [8] determined the optimal number of segments for the trunk when analysing walking and running. However, whether the approach presented by Kudo and colleagues will work on the other spine movements is unknown. Activities involving a wide motion of the spine are rarely studied. Back extension, for example, is an activity found in several sports, but little scientific information is available in the literature. Addressing these gaps in research could lead to an enhanced understanding of the kinematics and kinetics of the human spine, which could greatly improve injury prevention programmes.

Additionally, direct spinal kinematics and kinetics measurements are invasive and require complex procedures. The exact position of the vertebrae can be measured using imaging techniques such as fluoroscopy, ultrasound, MRI, CT scans, and X-rays [9–11]. However, the radioactive nature of some imaging techniques does not make it a viable option for all situations. Additionally, image-based techniques are captured only in a static or neutral position, complicating the spine dynamics analysis. Direct measurement of internal loading is usually measured in cadavers [12–15] due to the health risk issues they represent. However, cadaveric data might not be fully representative, as few specimens are available, and mechanical differences with living tissue may exist. Only a few studies have attempted to measure internal loading directly. Instruments, such as pressure transductors, have been inserted into spine tissues to obtain intervertebral pressure [16, 17]. Despite the clear risks to participants’ health, these results may serve as a reference point for future studies.

Optical motion capture systems are the current gold standard for human kinematics measurement. Optical motion capture systems consist of the visual tracking and triangulation of markers usually placed on participants’ skin. Furthermore, a good correlation between skin markers and the spinous process has been found [18, 19]. Recent review studies have addressed the measurement of spine motion using optical motion capture systems [20–23]. For instance, spine kinematics during specific activities such as gait [20] and sit-stand [21] have already been reviewed. Moreover, Negrini et al. [22] reviewed the techniques for assessing torso kinematics related to pathologies and marker location. Fayad et al. [23] reviewed the available literature regarding multi-segmentation of the thoracolumbar spine. On the other hand, Hesby et al. [24] researched the available technologies for measuring the motion of the cervical spine. Nevertheless, different movements, particularly in the torso region, can result in different outcomes depending on marker placement. Although several marker sets have been suggested for spine biomechanics, there is a lack of literature regarding the standardisation of the method. To date, no study has identified the optimal marker placement on the torso that may provide enough information on the spine alignment throughout movement [25]. The intervertebral rotation identification method could significantly alter the results and subsequent analysis, directly impacting the comparison between studies [26]. Therefore, understanding the current marker sets, segmentation approaches, and outcome parameters for analysing human spine dynamics is a step forward in standardizing the scientific literature.

This Scoping Review aims to summarise the methods and results that are available in the spine motion literature using optoreflective motion capture systems by providing information about used marker sets, reported results, type of spine segmentation (either uni-segmental or multi-segmental), and the studied activity. To find this information, the following research questions were addressed:

1. What marker sets are used to measure the kinematics of the spine in motion capture studies?
2. What protocols allow for a multi-segmental analysis of the spine?
3. What parameters can be measured from each used protocol?
4. In what activities has the motion of the spine been analysed?
5. What are the characteristics of the subjects measured in motion capture spine studies?

Through this article, the authors will refer to five sections, each addressing the research question, respectively: (1) marker sets, (2) segments, (3) measured parameters, (4) studied activities, and (5) participants. The authors intend to provide a general overview of spine motion studies, providing necessary information to the reader to select the marker location and model suitable for their study. Unlike other body structures, the torso (including the spine) has complex movements from respiration and the spine structure itself. Hence, the selected model and marker location could impact the obtained results.

## Methods

### Protocol

A Scoping Review design to analyse spine motion was completed by January 24, 2024, following the guidelines of the PRISMA extension for Scoping Reviews [27]. The protocol used to perform this Scoping Review was not registered. Eligibility criteria consisted of journal and conference articles (article type) on human spine motion using optical motion capture technologies (topic). Only articles written in English were included in the searches. Participant characteristics, such as gender, age, or ethnicity, were not considered for inclusion or exclusion. The articles were found in peer-reviewed, broad-spectrum, SCOPUS, and Web of Science databases. The main keywords for the search were ‘spine’ and ‘motion capture’, as the main subject of the study is the motion capture of the spine. Support keywords addressing brands and marker properties were used to refine the search. These include ‘reflective marker’, ‘Vicon’, ‘Optitrack’, and ‘Qualisys’. The search was performed directly on the database website (i.e. SCOPUS and Web of Science)

**Fig. 1:**
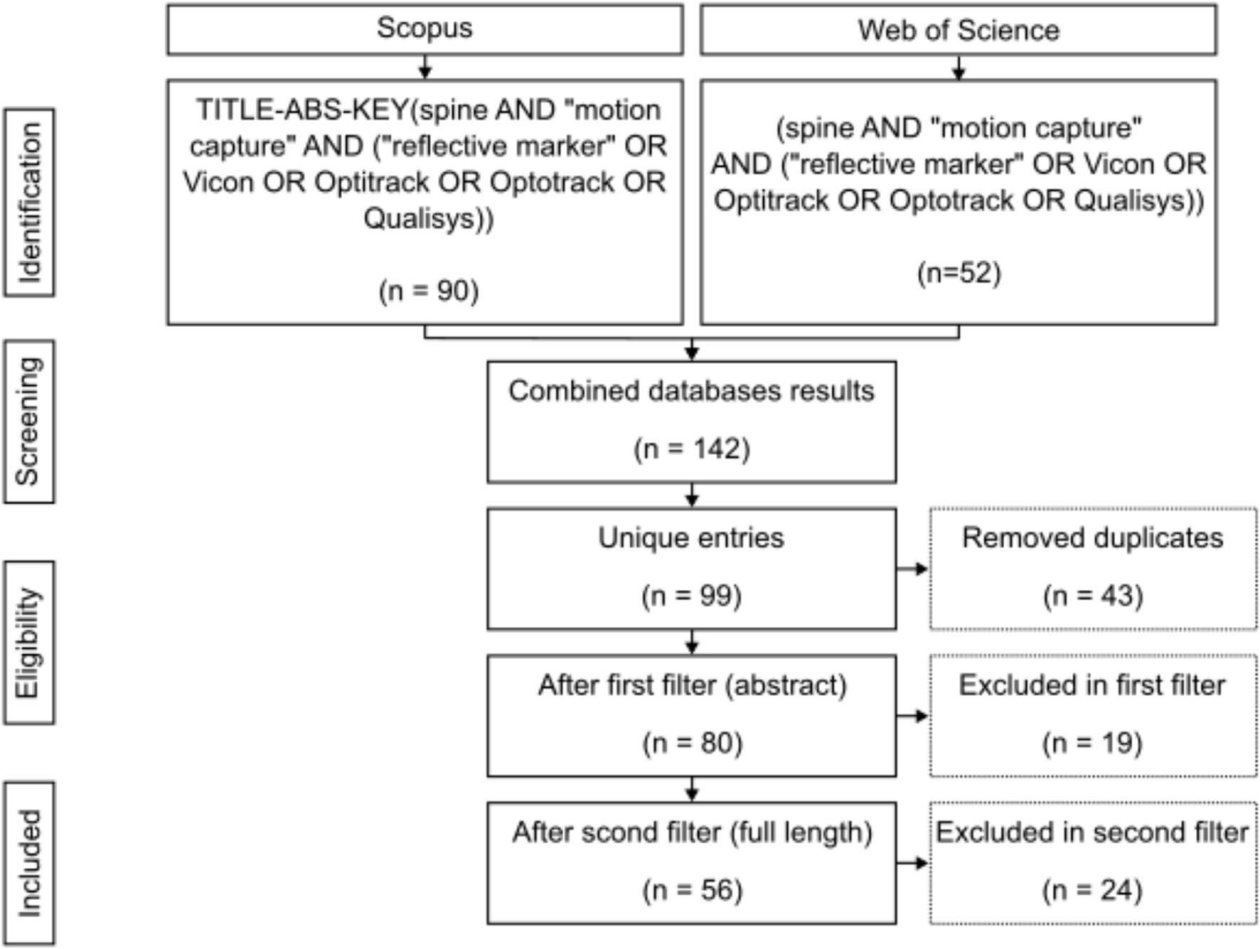
Flowchart summarising the process and resulting quantities from the article search. Search strings are equivalent for both databases

The resulting list of each database was exported and combined. Duplicates were removed using a custom MATLAB script. Next, the abstracts of the articles were carefully read as a first filter. During this step, articles that studied animals or cadavers were excluded. The resulting list was subjected to a second filter, where the articles were read in total length, thus obtaining the final list. Only articles using a marker-based motion capture analysis, specifying the marker placement, reporting spine results, and having the spine as an object of study were included in the final list. The search and refinement process for this Scoping Review is presented in Fig. 1.

### Data Extraction

Information such as used marker set, studied spine segments, modelling type, studied activities, reported results, and participant information were extracted from each included article. The name of the used marker set was recorded when available. This review considers the first three segments of the spine: cervical (C0 to C7), thoracic (T1 to T12), and lumbar (L1 to L5). The study did not consider the sacral segment and coccyx due to the difficulty of motion tracking.

This review uses three classifications for the segmentation type used in each article: (1) uni-segmental for the whole torso, (2) anatomical segments for studies of cervical, thoracic, or lumbar as segments, and (3) multi-segmental if any anatomical segment was broken down. Torso as a segment is considered from C7 to S1. All activities were labelled with general words, such as ‘walking’ and ‘running’, and divided into groups. Movement reported as anterior-posterior or happening in the sagittal plane was labelled as FE (flexion-extension), as lateral or happening in the frontal plane as LB (lateral bending), and rotational or happening in the transverse plane as AR (axial rotation). Frontal and coronal planes were considered the same. Participant age and gender were extracted when available. Participants were classified into three age groups: young under 18, adults between 18 and 60, and older adults over 60 years old.

## Results

### Selection of Sources

The initial search yielded 90 and 52 articles for Scopus and Web of Science, respectively. Following the refinement process, broken down in Fig. 1, the final list included 56 articles from 1997 to 2023. All included articles were classified by marker set, studied segment, and analysed activity (Table 1). Table 2 shows the relation between measured parameters, studied segments, and marker sets. Figure 3 presents the number of articles analysing specific activities. Participant characteristics are summarised in Table 3.

**Table 1:** Studied motions found in the review, grouped by used marker set and studied segments

Marker set	Studied segments	Studied motion
Crewe	Lumbar	Lifting [28]^a^
IfB	Lumbar	Sit & stand, lifting, walking, running, jumping [1]^a^
	Thoracic & lumbar	Standing [29]^a^ [30]^b^
		Walking [29]^a^
Plug-in-Gait	Cervical	Walking [37]^ab^
	Lumbar	Cart [38]^a^
		Lifting [39]^a^
	Torso	Running, kicking [32]^a^
		Standing [34]^a^ [36]^c^
		Walking [31]^c^ [33]^a^ [34]^a^ [36]^c^
		Throwing [35]^a^
	Cervical & thoracic	Hockey-shoot [40]^a^
	Cervical & torso	Yoga [41]^a^
	Thoracic & torso	Walking [42]^a^
Plug-in-Gait & IfB	Thoracic & lumbar	Walking [43]^ab^
Plug-in-Gait modified	Lumbar	Jumping [46]^ab^
		Walking, kneeling, sit&stand, lay&stand, stairs, lifting [48]^b^
	Torso	rowing [45]^a^
	Lumbar & torso	FE, LB, AR, handling [49]^a^
	Thoracic & lumbar	Handling [47]^a^
		Sitting [44]^a^
Ranavolo	Cervical & thoracic	FE [50]^a^
Mesh	Thoracic	FE, LB, AR [51]^a^
		Running [52]^d^
Other	Cervical	FE, LB, AR [64]^ac^
		Walking [65]^d^
	Thoracic	Standing [66]^a^
	Lumbar	FE [53]^a^ [56]^a^ [58]^a^ [60]^ab^ [61]^a^
		LB [60]^ab^
		AR, lunging [56]^a^
		Walking [55]^a^ [56]^a^ [63]^a^
		Bowling [59]^a^
		Sit & stand [57]^ab^
		Standing [55]^a^ [63]^a^ [82]^a^
		wheelchair [62]^a^
	Torso	Standing, FE, LB, AR [67]^a^ [69]^a^
		Walking [68]^a^ [69]^ab^ [70]^b^
		Running [68]^a^
	Cervical & torso	FE, LB [78]^d^
	Lumbar & torso	Sit & stand, lunging, squat, walking, standing [80]^a^
	Thoracic & lumbar	AR [73]^a^ [75]^b^
		FE [72]^a^ [73]^a^
		LB [73]^a^
		Running [77]^acb^
		Standing [2]^a^ [74]^a^ [77]^abc^
		Walking [71]^a^ [76]^ab^
		Sit & stand, lifting [76]^ab^
	Thoracic, lumbar & torso	Walking [81]^a^
	Thoracic & torso	FE, LB, AR [79]^a^

^a^Healthy participants

^b^Participants suffering a spine pathology

^c^Participants underwent a spine surgery

^d^No participant information

**Table 2:** Reported measurements found in the review, grouped by used marker set and studied segments

Marker set	Studied segments	References	Reported measured parameters				
			Angles	Angular velocity	Curvature	Linear kinematics	Kinetics
Crewe	Lumbar	[28]^am^	FE				
IfB	Lumbar	[1]^a^			Sagittal		
	Thoracic & lumbar	[29]^a^	FE, LB & AR		Sagittal		
		[30]^a^			Sagittal		
Plug-in-Gait	Cervical	[37]^a^	FE, LB & AR				
	Cervical & thoracic	[40]^ua^	AR				
	Cervical & torso	[41]^ua^	FE				
	Lumbar	[39]^a^					Compression
		[38]^m^					Compression & shear
	Thoracic & torso	[42]^ua^	FE, LB & AR				Compression & momentum
	Torso	[31]^ua^	FE				
		[32]^u^	FE & AR				
		[33]^u^	AR				
		[34]^u^				Position	
		[35]^u^	FE, LB & AR				
		[36]^u^	FE, LB & AR				
Plug-in-Gait & IfB	Thoracic & lumbar	[43]^a^	FE, LB & AR		Sagittal		
Plug-in-Gait modified	Lumbar	[48]^a^	FE & LB				
	Lumbar	[46]^a^	FE		Sagittal		
	lumbar & torso	[49]^ua^	FE, LB & AR				
	Thoracic & lumbar	[47]^a^	FE, LB & AR				
		[44]^m^				Position	
	Torso	[45]^u^	FE				
Ranavolo	Cervical & thoracic	[50]^a^		FE			
Mesh	Thoracic	[51]^m^	FE				
		[52]^m^	FE, LB & AR				
Other	Cervical	[64]^a^	FE, LB & AR	FE, LB & AR			
		[65]^a^	FE				
	Cervical & torso	[78]^ua^	FE, LB & AR				
	Lumbar	[53]^am^	FE				
		[54]^a^			Sagittal		
		[55]^m^	FE				
		[60]^ua^	FE & LB				
		[56]^a^	FE & AR				
		[63]^a^	FE				Compression
		[57]^a^			Sagittal		
		[58]^a^	FE, LB & AR				
		[61]^am^	FE				
		[59]^a^	FE, LB & AR				Compression, shear & momentum
		[62]^a^				Position	
	Lumbar & torso	[80]^ua^	FE			Position	
	thoracic	[66]^m^				Acceleration	
	thoracic & lumbar	[2]^am^			Sagittal		Compression
		[72]^am^	FE		Sagittal		
		[77]^m^	FE				
		[73]^a^	FE, LB & AR				
		[74]^a^					
		[75]^m^	AR				
		[76]^a^	FE			Position & velocity	
		[71]^m^	AR				
	Thoracic, lumbar & torso	[81]^ua^	FE & LB		Frontal		
	Thoracic & torso	[79]^m^	FE, LB & AR				
	Torso	[67]^u^	FE, LB & AR				
		[68]^u^	LB & AR			Position	
		[69]^u^	FE, LB & AR				
		[70]^u^	FE				

*FE* Flexion-extension, *LB* lateral bending, *AR* axial rotation

^u^uni-segmental studies

^a^anatomical segment studies

^m^multi-segmental studies

**Table 3:** Participant age and gender groups

Gender	Age group			Total
	young (< 18 years)	Adult (18–60 years)	Older adults (> 60 years)	
Male	43	345	55	443
Female	103	265	97	465
Not specified	12	59	0	71
Total	158	669	152	979

### Summary of Outcome Measures and Findings Across Study Themes

#### Marker Sets

**Fig. 2:**
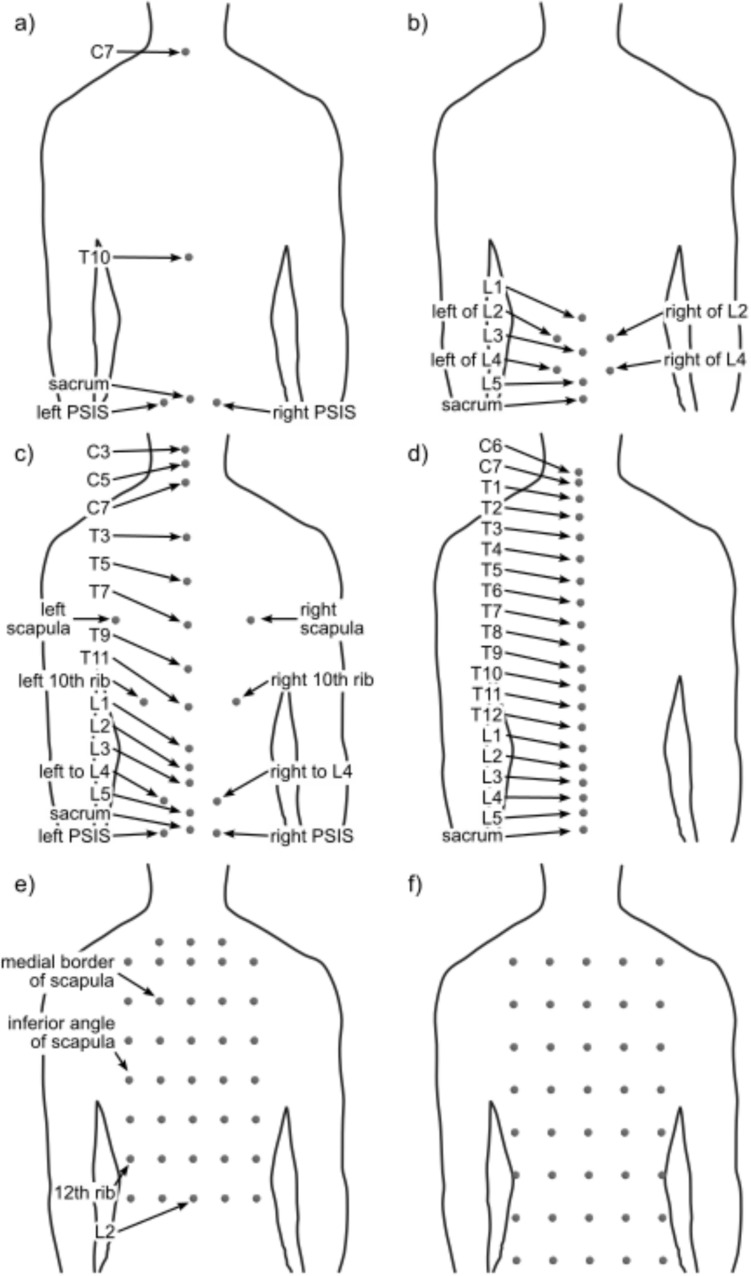
Comparison of previously defined marker sets identified in the review, back view: **a** Plug-in-Gait marker set; at the front markers placed at notch xiphoid and both ASIS; **b** Crewe marker set; at the front markers placed at both ASIS; **c** IfB, marker set, additional markers placed at the tip of the iliac crest, sternum and both ASIS; **d** Ranavolo marker set; **e** mesh strategy using anatomical position as anchors to create a 8-row 5-column mesh in the front and back, and one column of five markers at both sides; and **f** mesh strategy evenly placed on the torso

Four marker sets were identified: Crewe, IfB, Plug-in-Gait, and Ranavolo. Marker set configurations are compared in Fig. 2. One article (1.79%) used the Crewe marker set [28]. Three articles (5.36%) used the IfB marker set alone [1, 29, 30]. Twelve articles (21.43%) used the Plug-in-Gait marker set [31–42]. One article (1.79%) used a combined marker set between Plug-in-Gait and IfB [43]. Six (10.71%) a modified version of the Plug-in-Gait marker set [44–49]. One article (1.79%) used the marker set proposed by Ranavolo [50]. Additionally, two studies (3.57%) placed markers in a mesh-like array along the torso by using specific bony landmarks as reference [51] and assigning a specific vertical and horizontal distance between markers [52]. Finally, 30 articles (53.57%) used a non-specified marker set [2, 53–81]. Table 1 summarises the articles included in the review grouped by marker set, studied segment, and studied activity.

#### Segments

Table 2 shows the type of segmentation each article used as a super index in the reference column. Twenty-one articles (37.5%) used anatomical segmentation [1, 29, 30, 37, 39, 43, 46–48, 50, 54, 56–59, 62–64, 73, 74, 76]. Ten studies (17.86%) analysed the thoracolumbar spine as a segment [32–36, 45, 67–70]. Ten (17.86%) multi-segmental studies were found [38, 44, 51, 52, 55, 66, 71, 75, 77, 79]. Nine articles (16.07%) performed an anatomical and uni-segmental segmentation [31, 40–42, 49, 60, 78, 80, 81], and five (8.93%) did an anatomical and multi-segmental analysis [2, 28, 53, 61, 72].

#### Measured Parameters

Measured spine parameters obtained via motion capture were grouped into five categories: angular kinematics, including angles and angular velocity, curvature, linear kinematics, and kinetics (see Table 2). Angles and angular velocities correspond to the spinal position in either of the three directions (FE, LB, and AR). Thoracic and lumbar curvatures were identified to be reported in the sagittal and frontal planes. Overall angles in the three planes were the most reported measurements. Forty articles (71.43%) reported angular measurements [28, 29, 31–33, 35–37, 40–43, 45–49, 51–53, 55, 56, 58–61, 63–65, 67, 69, 71–73, 75–80]. Thirty-five articles (62.5%) reported FE angles [28, 29, 31, 32, 35, 37, 41–43, 45–49, 51–53, 55, 56, 58–61, 63–65, 67, 69, 72, 73, 76–80], fifteen (26.79%) LB [35, 37, 42, 43, 47–49, 58–60, 64, 67, 69, 73, 78], and twenty (35.71%) AR [32, 35, 37, 42, 43, 47, 49, 56, 58, 59, 64, 67, 69, 73, 78]. Just two studies (3.57%) reported angular velocity [50, 64], nine (16.07%) curvature in either sagittal or frontal plane [1, 29, 30, 43, 46, 54, 57, 72, 81], seven (12.5%) any linear kinematics [34, 44, 62, 66, 68, 76, 80], and six (10.71%) kinetic measurements [2, 38, 39, 42, 59, 63]. Only Plug-in-Gait and IfB marker sets were used to calculate curvature.

#### Activities

**Fig. 3:**
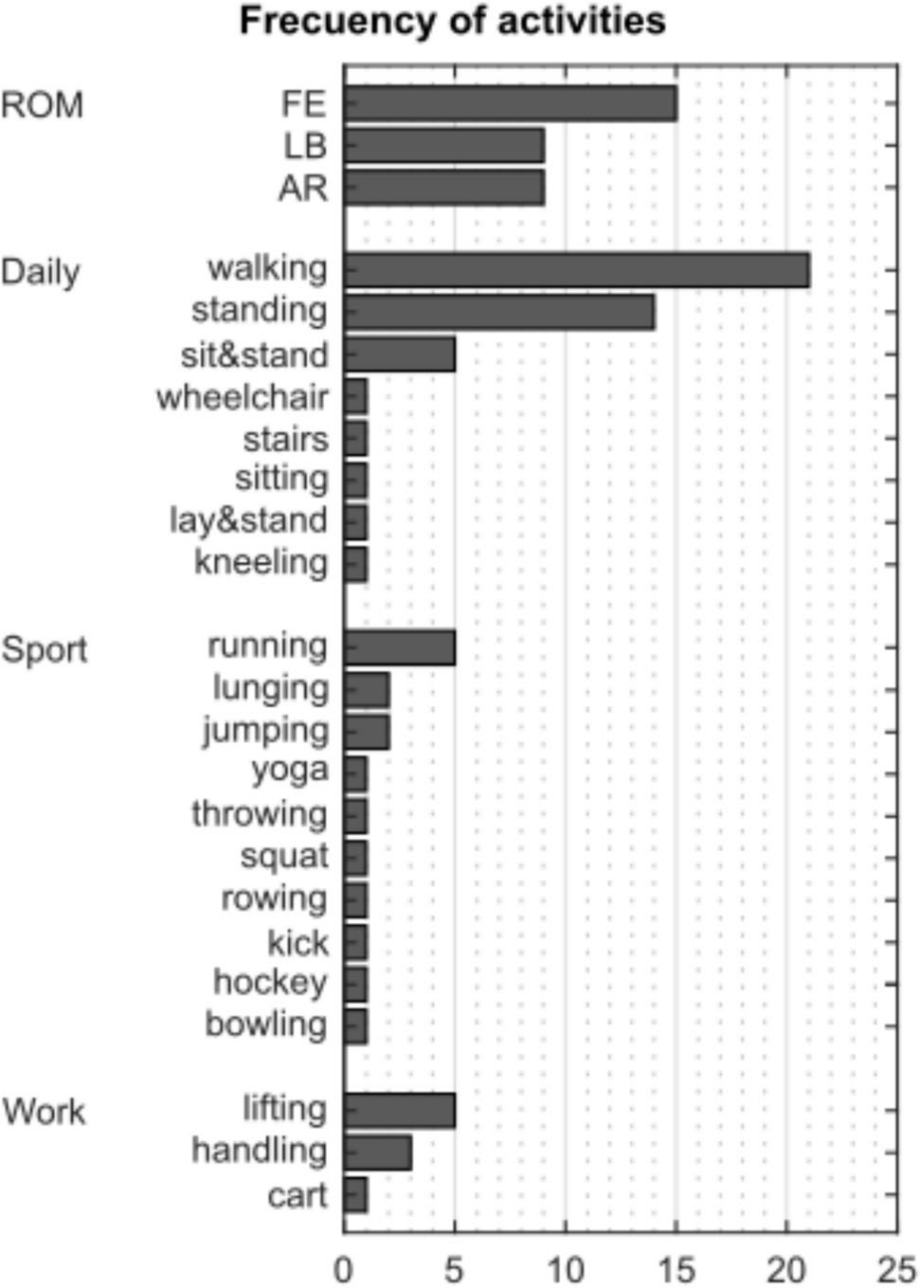
Frequency of studied activities identified across included articles

Activities were grouped into four classifications (ROM, daily, sport, and work) and twenty-five sub-classifications, generally describing the studied activity (Fig. 3). Thirty-three articles (58.93%) studied ROM: fifteen (26.79%) FE [49–51, 53, 56, 58, 60, 61, 64, 67, 69, 72, 73, 78, 79], nine (16.07%) LB [49, 51, 60, 64, 67, 69, 73, 78, 79], and nine (16.07%) AR [49, 51, 56, 64, 67, 69, 73, 75, 79]. Forty-five studies (80.36%) were performed in daily activities [1, 2, 29–31, 33, 34, 36, 37, 42–44, 48, 54–57, 62, 63, 65–71, 74, 76, 77, 80, 81], from which walking (37.5%) [1, 29, 31, 33, 34, 36, 37, 42, 43, 48, 55, 56, 63, 65, 68–71, 76, 80, 81], standing (25%) [2, 29, 30, 34, 36, 54, 55, 63, 66, 67, 69, 74, 77, 80], and sit&stand (8.93%) [1, 48, 57, 76, 80] activities were the most popular. Sport- and work-related activities were studied by sixteen (28.57%) [1, 32, 35, 40, 41, 45, 46, 52, 56, 59, 68, 77, 80] and nine (16.07%) [1, 28, 38, 39, 47–49, 76], respectively. Walking was the most studied activity (in twenty-one articles 37.5%), followed by FE (in 15 articles 26.79%) and standing (in 14 articles 25%).

#### Participants

Nine hundred seventy-nine participants were recruited for the reviewed studies (Table 3). Most of the recruited participants were adult participants (345 males 35.24%, 265 females 27.07%, 59 not specified 6.03%), followed by young (43 males 4.39%, 103 females 10.53%, 12 not specified 1.23%) and older adults (55 males 5.62%, 97 females 9.91%). The participant age ranged from 7.4 to 91 years across all articles.

## Discussion

### Principal Findings

#### Marker Sets

Plug-in-Gait was the most common marker set as expected. It considers four posterior markers, two anterior, and five pelvic (Fig. 2a). A modified version of the Plug-in-Gait was also common and usually replaced the T10 marker with T12. However, the Plug-in-Gait marker is rarely used for multi-segmental analysis and is instead used for studies of the torso (uni-segmental) or anatomical segments. Only two articles attempted to perform a multi-segmental analysis to calculate the T10 position [44] and compression between L4 and L5 [38]. Crewe and Ranavolo marker sets were used in only one article, respectively, both analysing anatomical segments. Crewe marker set was developed to analyse the lumbar spine and reported multi-segmental results [28]. Ranavolo marker set was used to compare and evaluate an IMU-based motion capture system for a cervicothoracic analysis [50].

For a better understanding of spine motion, more complete marker sets might bring better results, especially when considering more significant curvature changes. Glover et al. [25] concluded that more markers on the spine have a better performance regardless of the location when studying running. Therefore, IfB and Ranavolo marker sets might be more accurate in investigating the motion of the spine, allowing for multi-segmental studies. However, despite having several markers placed among the spine (Fig. 2c and d), IfB and Ranavolo marker sets were not used for multi-segmental analyses.

Both articles reporting a meshing strategy (Table 1 and Fig. 2e and f) studied thorax deformation rather than the spine motion but reported angles of a section of the spine. It is worth mentioning that both studies used different strategies when deciding the location of the marker. One article used anatomical landmarks to specify the distance between markers in horizontal and vertical directions [51]. The second article evenly distributed the markers in eight rows and five columns on the back of the participants [52].

During kinetic predictions, the creation of virtual reference frames is desired. At least three non-linear markers are needed to create three orthogonal axes. Hence, IfB or Crewe marker sets might be more beneficial for kinetic analysis, as they include lateral vertebral markers. However, the reported results do not support this idea, as neither was it used to predict kinetic measurements. A comparison between existing marker sets has yet to be made. Therefore, a study of this nature will help the scientific community to decide which marker set might be more suitable for any specific application.

#### Segments

The lumbar area is the most common subject of study. It has more mobility than the thoracic spine and supports more weight than the cervical and thoracic spine. Low back pain is a condition affecting the lumbar spine in many people. In severe cases, low back pain could impact the lives of patients, causing disability, functional limitations, and retirement [83–85]. All articles using IfB and Crewe marker sets analysed the lumbar segment. However, most articles studying this segment did not use a named marker set. The articles that studied the lumbar segment with the Plug-in-Gait marker set made modifications for this purpose. Hays et al. [48] switched the marker at T10 to T12 to better segment the lumbar spine. Ghasemi et al. [49] added T1, T12, and S1 markers. Movahed et al. [46] added T12 and L3 to include the lumbar spine in their study.

Unexpectedly, the cervical spine was the least studied segment, although it is very mobile, and neck pain has become the second most common musculoskeletal pathology after low back pain [86]. A possible reason could be found in the review of Hesby et al. [24], where optoelectronic systems are not the most prevalent measuring systems for cervical kinematics. Other options include photogrammetry, 2D and 3D electro-goniometers, inclinometers, ultrasound, and magnetometers. Several works have studied kinematics in patients with neck pain [4, 64] and kinematics in cervical injuries [87].

Plug-in-Gait and its variants were mainly used primarily for uni-segmental or thorax analysis. Anatomical analyses were common, with thirty-one out of 56 reviewed articles including cervical, thoracic, and lumbar segments. Multi-segmental studies always included the thoracic and lumbar spine but never the cervical spine. Meshed-like marker sets were used to perform this type of analysis. The Crewe marker set was also used, as it was created to address a multi-segmental approach. A modified version of the Plug-in-Gait marker set, with additional markers on T3 and T4, measured the position of the T10 vertebra [44]. Mullerpatan et al. [41] studied cervical flexion during yoga. And Asadi et al. [47] studied the thorax and lumbar angular kinematics while handling several objects. Despite being widely used across studies, Plug-in-Gait might not have enough resolution to identify spine segments correctly.

#### Measured Parameters

Across all articles, angular kinematics (especially angles) was the most reported parameter. As expected, FE is the motion that raises more curiosity, as it usually represents the broader range of movement and is involved in most studied activities (e.g. sitting and lifting). Angular velocity in LB and AR was only measured in one article [64]. No angular acceleration was reported. Linear kinematics was poorly documented, mostly reporting the position of any specific vertebrae. Unexpectedly, curvature parameters are somehow scarce. The IfB marker set was always used for curvature calculations.

Only some articles attempted to calculate kinetic parameters. Compression force was the most reported kinematic result. Five articles reported compression calculated on the lumbar segment [2, 38, 39, 59, 63]. Marker-based motion capture systems alone cannot calculate kinetic parameters. Usually, joint internal forces and loading are calculated using computer musculoskeletal models, driven by marker coordinates through inverse dynamic algorithms. Reaction forces may also be captured to provide more information for the computational model. The contribution of muscle forces and external loads should be considered to properly analyse injury mechanisms [87]. Internal forces and momentum are crucial to understanding injury mechanisms, especially for overuse injuries [3]. Glover et al. [25] studied the effects of tracking spine motion in a computerised model with and without motion between the vertebrae during running. Beaucage-Gauvreau et al. [88] validated an OpenSim full-body model with six lumbar segments to estimate spinal loading during scoop, squat, and one-handed lifting tasks. Spinal loads predicted by models are sensitive to analysed motion and errors in the orientation of the thorax and pelvis [9].

Nevertheless, the reported parameters of motion capture are indirect measurements. Few in vivo studies have attempted to capture the direct motion of the vertebrae. In this review, only one article performing direct calculations was identified. MacWilliams et al. [55] used bony pins to attach marker clusters directly to the spinous processes of L1 to S1. Despite the emphasis on the procedure’s invasiveness, their subjects reported only mild discomfort. Still, their results in FE, LB, and AR while walking are valuable as they measure the position of the vertebral bones directly.

#### Activities

Overall, ROM activities are the most studied across the articles included in this review (Fig. 3). This was unexpected, as ROM exercises allow control and movement isolation in a single plane. ROM has been used for most pathological studies to understand injury mechanisms or alterations in movement patterns. Daily activities are the second most common subject of study. The fact that most sports and work-related activities have few studies might result from difficulty measuring spine motion. This result clearly shows the need for the scientific community to find better strategies to measure spine motion to address complex movements. On the other hand, the Plug-in-Gait marker set has been used to study a variety of activities. Since the Plug-in-Gait marker set was designed for walking, caution is recommended while using this marker set, as it may not be accurate when used in other activities where the spine curvature changes significantly [44], e.g. in acrobatic sports, where athletes are encouraged to develop back flexibility.

#### Participants

Both genders were widely studied across the included articles (Table 3). However, the difference for the younger population is considerable for both genders (43 males vs 103 females). The reason was found to be four articles studying idiopathic scoliosis [30, 36], c1-c2 subluxation [37], and spine position [34] in young females. The first condition primarily affects young females. However, measuring females can be challenging due to the difficulty of exposing the torso. The best approach might involve female participants wearing a sports bra during measurements. Hence, some vertebral markers might be placed on the fabric in complex marker sets, resulting in more movement between the marker and the bone.

### Knowledge Gaps

With the current information in the literature, it is challenging to determine how many segments are suitable for analysing the human spine for any specific activity. Kudo et al. [8] concluded that segmenting the torso into two or three parts gives enough information to study spine motion during running and walking. However, whether this approach suits other activities is yet to be known. Most of the included articles failed to report the mathematical approaches applied to calculate the results. Because of the geometrical nature of the procedures, mathematical decisions may directly affect the obtained data. For example, the rotations’ order directly affects the resulting anatomical angles [89]. Additionally, coordinate system creation is sensitive to the location and number of markers used. The authors encourage future studies to explicitly report mathematical decisions, especially while analysing spine motion in multiple planes, to allow for comparison between studies.

Daily and ROM activities are widely studied in the current literature. Due to impacts and external weight manipulation, sports and work-related activities may represent higher injury risk. However, these activities are underinvestigated. As high forces affecting the spine have also been reported to cause spine injuries, with sensitivity in the loading patterns [90], practitioners must understand the possible injury mechanisms to create successful rehabilitation or injury prevention programmes. Moreover, low back pain is a common pathology that affects the lifestyle of many people around the world. It is suggested that posture and ROM are essential factors to consider when analysing the origin of pain [6]. Evidence suggests movement inhibition could be both a cause and a consequence of experiencing low back pain [6]. High forces affecting the spine have also been reported to cause spine injuries, with sensitivity in the loading patterns [90]. In specific, the high repetition of FE movements combined with high-loading patterns has been reported to be the mechanism of spinal injuries in gymnasts [91]*.*

### Limitations

Biases in the study outcomes may be present due to limitations imposed by the databases utilized, eligibility, inclusion, and exclusion criteria, and search criteria input to identify studies of interest. In particular, a larger dataset could be constructed through the inclusion of medicine-oriented databases such as PubMed, PubMed Central, Cochrane Library, and EMBASE, as well as incorporating publications beyond the English language. Nevertheless, the fifty-six studies analysed in this report demonstrate clear trends indicating the importance of activity-oriented decision-making when addressing the kinematics and kinetics of the spine.

## Conclusion

This study presented a Scoping Review of the motion capture techniques and measurement outcomes of biomechanical studies of the human spine, in an effort to help with the standardization of the field. The results revealed that the Plug-in-Gait is the preferred marker configuration even though it has few markers on the spine. Nevertheless, caution is recommended when analysing other activities, as this marker set was developed for walking [44]. Although IfB and Ranavolo placed several markers on the spine, they were not used for a multi-segmental analysis. A mesh-like marker set helps examine torso deformation. The existent marker sets have yet to be compared, to understand in more depth the benefits of each marker set applied in specific activities. The lumbar spine is the segment that arises more interest as it is the base of the trunk and the most affected area since it is where lower back pain happens. The relationship between the thoracic and lumbar spine is also widely studied. However, marker-based motion capture systems have poorly studied the cervical spine. Angular position is the most common searched outcome when studying the spine, especially in the sagittal plane, where FE happens. Daily activities raise the most interest, particularly walking, which was the most studied among all activities. Studies on sports and work-related activities are scarce, even though these activities might inherit a higher injury risk. Moreover, there is a need to research spine posture and movement in more realistic scenarios. Finally, the adult population is widely studied. However, more studies of young and older adult participants are needed since several conditions and pathologies might affect the spine differently at various ages.

## Data Availability

Not applicable.
